# Transcatheter and Surgical Aortic Valve Replacement in Patients With Previous Cardiac Surgery: A Meta-Analysis

**DOI:** 10.3389/fcvm.2020.612155

**Published:** 2021-02-10

**Authors:** Yi-ming Li, Jia-yu Tsauo, Kai-yu Jia, Yan-biao Liao, Fan Xia, Zheng-gang Zhao, Mao Chen, Yong Peng

**Affiliations:** ^1^Department of Cardiology, West China Hospital, Sichuan University, Chengdu, China; ^2^Department of Neurosurgery, West China Hospital, Sichuan University, Chengdu, China

**Keywords:** transcatheter aortic valve replacement, aortic stenosis, previous cardiac surgery, meta-analysis, surgical aortic valve replacement

## Abstract

**Background:** Many patients who have aortic stenosis and are transcatheter aortic valve replacement (TAVR) candidates have underwent prior cardiac surgery (PCS). The aim of this study was to provide a robust summary comparison between patients with PCS who underwent TAVR vs. surgical aortic valve replacement (SAVR).

**Methods:** We conducted a systematic review and meta-analysis of all published articles on PubMed/Medline, Ovid, EMBASE, and Scopus from 2002 to 2019.

**Results:** A total of 13 studies were finally included, yielding a total of 23,148 participants. There was no statistical difference with 30-day [OR: 1.02 (0.86–1.21)] or 1-year mortality [OR: 1.18 (0.86–1.61)] between the two groups. Subgroup analysis revealed that high-risk patients who underwent TAVR with the transapical approach were associated with increased risk of mortality [OR: 1.45 (1.00–2.11)]. However, those who underwent TAVR with endovascular approach had a comparable outcome with SAVR.

**Conclusions:** Primary outcomes after endovascular TAVR were similar to those with SAVR and superior to transapical TAVR treatment group in patients with PCS.

## Introduction

Transcatheter aortic valve replacement (TAVR) and surgical aortic valve replacement (SAVR) are currently the main treatment options for high-risk patients with aortic stenosis (AS) (1, 2). Recent trials had demonstrated that TAVR has similar 2-year mortality outcome compared with SAVR in patients with intermediate surgical risk (3). In other large registries, Gleason et al. and Fraccaro et al. also found a similar trend at long-term follow-up (4, 5). Nevertheless, in those with prior cardiac surgery (PCS), the outcome difference between TAVR and SAVR remains controversial.

In PARTNER IA and PARTNER IIA studies, both TAVR and SAVR had shown comparable outcomes in patients with PCS (6, 7). Similarly, a separate study which focused on those who underwent TAVR with the transapical approach reported comparable short-term mortality rates with SAVR (Onorati et al.). In the CoreValve High Risk (CHR) study, however, it was revealed that TAVR was associated with significant morbidity advantage and improved survival compared with SAVR (8). On the other hand, some studies suggested that TAVR was associated with higher mortality compared with SAVR. Therefore, the purpose of this study was to investigate the outcomes of PCS patients between TAVR vs. SAVR. In addition, we sought to pool the multivariate outcomes of important relevant endpoints, as well as subgroup analysis with risk classification and access approach to provide a robust summary conclusion.

## Methods

**Figure 1:**
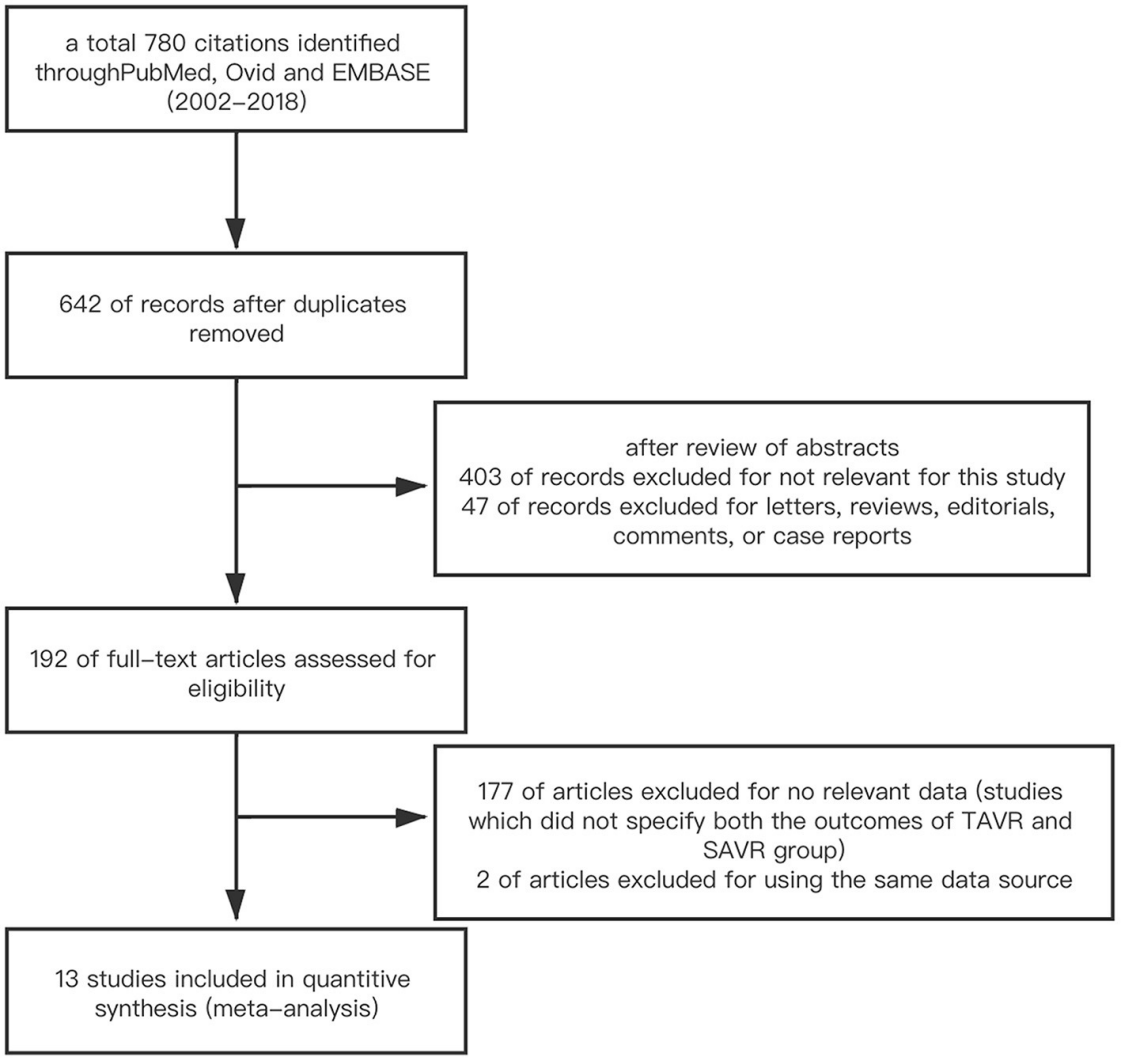
Flow diagram of study selection.

We conducted a literature search on PubMed/Medline, Ovid, EMBASE, and Scopus (2002–2019). The search terms were as follows: transcatheter aortic valve implantation; transcatheter aortic valve replacement; surgical aortic valve replacement; previous cardiac surgery; prior coronary artery bypass surgery; previous valve surgery. Multi-step assessment was performed to identify the articles qualified for this meta-analysis (Figure 1). Inclusion criteria were studies which reported the outcomes of TAVR vs. SAVR in those with PCS. Studies were excluded based on at least one of the following: (1) studies which were published in the form of letter, review, editorial comment, or case report; (2) studies which did not specify both the outcomes of TAVR and SAVR group; (3) non-English language study. If duplicate data source occurred, the one with the largest sample size was included to avoid duplicate publication.

The definition of PCS was utilized according to the primary articles. Sensitivity analysis would be performed with the exclusion of studies which included patients with surgical history for aortic valve. The primary endpoints of this meta-analysis were short-term (30-day), mid-term (1-year), and overall follow-up all-cause mortality. The secondary endpoints were stroke, bleeding, acute kidney injury, and new permanent pacemaker implantation (PPMI) during the period of hospital stay, as well as follow-up.

Two authors (Y.L. and J.T.) extracted the data independently, including author names, regions, publishing years, number of cases, patients' baseline characteristics including age, gender, transapical (TA) approach, and endovascular (EV) approach, which includes transfemoral, transaxillary, and transcarotid approach, logistic European System for Cardiac Operative Risk Evaluation (EuroSCORE), the Society of Thoracic Surgeons (STS) score, and the length of follow up. The Newcastle–Ottawa scale was performed to assess the quality of each included studies. In addition, subgroup analyses was performed to investigate the impact of different access approach and surgical risk (intermediate risk: STS score 4–8% or logistic EuroSCORE 10–20; high risk: STS score >8% or logistic EuroSCORE >20) on the mortality outcome.

Results of categorical variables are presented as n% and continuous variables are expressed as the mean ± SD. The inverse variance method was utilized to pool the OR and HR. For heterogeneity, it would be considered significant if the *p* < 0.05 and the *I*^2^ statistic was >50%. The DerSimonian and Laird random-effect methods were performed when significant heterogeneity was observed between the studies. All statistical analysis was conducted by Stata MP software version 14.2 (StataCorp LLC, 4905 Lakeway Drive, USA).

## Results

A total of 780 citations were initially identified. After deleting duplicate publications, we performed a multi-stage assessment based on the literature title, abstract, and then careful full-text review. Finally, 13 studies including 23,148 participants were included in this systematic review and meta-analysis (6–18). The process of study selection is summarized in Figure 1. The baseline characteristics of each included studies are summarized in Table 1.

**Table 1:** Baseline characteristics of the included studies.

**Authors**	**Published year**	**Design**	**TAVR (*n*)**	**SAVR (*n*)**	**EV vs. TA (*n*, %)**	**Follow-up**	**Log EuroSCORE (%)**	**STS Score (%)**
Stortecky S	2011	Single center	40	40	29, 62.5% vs. 11, 27.5%	TAVR: mean 274 ± 201 daysSAVR: mean 659 ± 464 days	TAVR: 33.5 ± 17 SAVR: 20.2 ± 14	TAVR: 7.6 ± 7SAVR: 6.3 ± 6
Jegaden O	2012	Single center	13	10	4, 30.8% vs. 9, 69.2%	1 year	25 ± 15	–
Wilbring M	2013	Single center	53	53	0, 0% vs. 53, 100%	Mean 245 ± 323 days	TAVR: 29.9 ± 14.0 SAVR: 26.4 ± 12.9	
Papadopoulos N	2014	Multi center	40	40	0, 0% vs. 40, 100%	4 years	TAVR: 24 ± 6 SAVR: 19 ± 6	TAVR: 11.1 ± 2.8SAVR: 10.4 ± 3
Nguyen TC	2014	Single center	107	148	51, 47.7% vs. 56, 52.3%	2 years	–	9.1 ± 6.4
Greason KL	2014	PARTNER IA	148	140	–	2 years	TAVR: 34.6 ± 16.8 SAVR: 33.8 ± 15.3	TAVR: 11.8 ± 3.3SAVR: 12.0 ± 3.1
Scherner M	2014	Single center	77	59	0, 0% vs. 77, 100%	3 years	TAVR: 24.99 SAVR: 20.68	TAVR: 11.2 ± 4.3SAVR: 9.9 ± 3.3
Wendt D	2015	Single center	62	51	–	1 year	TAVR: 36.4 ± 17.4 SAVR: 22.2 ± 17.5	TAVR: 12.1 ± 10.0SAVR: 7.1 ± 5.2
Conte JV	2016	CHR study	115	111	115, 100% vs. 0, 0%	1 year	TAVR: 25.6 ± 16.2 SAVR: 24.2 ± 15.8	TAVR: 7.3 ± 2.7SAVR: 8.0 ± 3.5
Reinöhl J	2016	Multi center	4,194	2,027	–	In hospital	TAVR: 33 ± 15 SAVR: 15 ± 10	–
Onorati F	2016	ITA Registry and RECORD Registry	28	28	0, 0% vs. 28, 100%	TAVR: mean 12.2 monthsSAVR: mean 20.1 months	–	–
Gupta T	2018	NIS database: 2012–2014	8,885	6,170	7,005, 78.8% vs. 1,880, 21.2%	In hospital	–	–
Chen S	2018	PARTNER 2A	245	264	188, 76.7% vs. 57, 23.3%	2 years	10.4 ± 8.4	6.1 ± 2.0

*NIS, National Inpatient Sample; TAVR, transcatheter aortic valve replacement; SAVR, surgical aortic valve replacement; EV, endovascular; TA, transapical; EuroSCORE, European System for Cardiac Operative Risk Evaluation; STS, the Society of Thoracic Surgeons Cardiac Surgery Risk Models; CHR, CoreValve High Risk study*.

### Study Quality and Risk of Bias Assessment

The quality assessment was performed by Newcastle–Ottawa Scale (http://www.ohri.ca/programs/clinical_epidemiology/oxford.htm). All of the included studies were of high quality (>6). The symmetry of the funnel plot and Egger's test on the outcomes indicated that there were no publication bias in the included studies.

### Follow-Up Outcome

**Figure 2:**
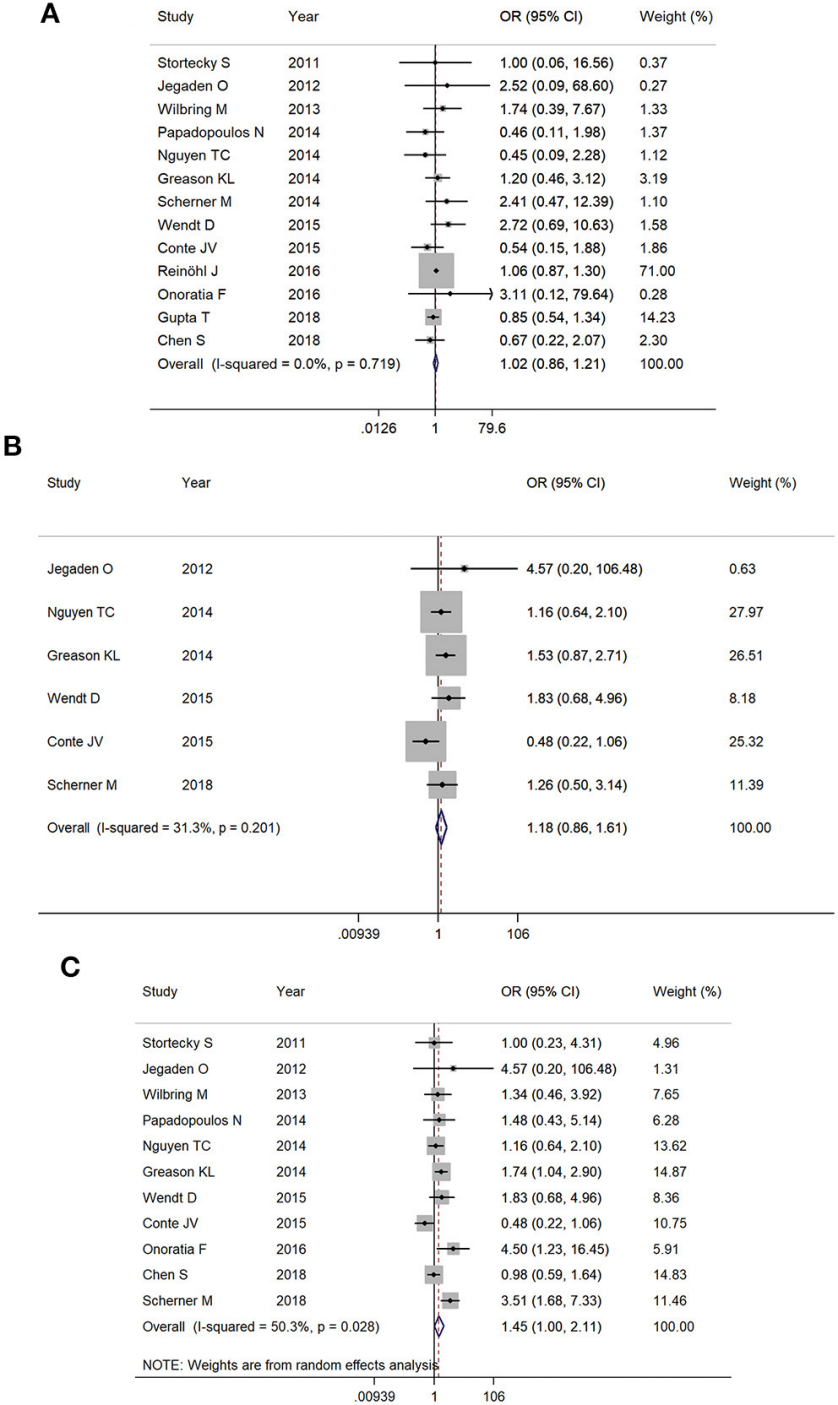
The meta-analysis for **(A)** 30-day mortality, **(B)** 1-year mortality, and **(C)** overall follow-up mortality.

All-cause mortality within 30 days was 3.9% (553/14,007) in the TAVR group and 3.7% in the SAVR group (338/9,141) (OR 1.02, 95% CI 0.86–1.21, *I*^2^ = 0%) (Figure 2A). At 1 year, the mortality rate was 19.9% (104/522) in the TAVR group and 17.9% (93/520) in the SAVR group (OR 1.18, 95% CI 0.86–1.61, *I*^2^ = 31.3%) (Figure 2B). Based on 10 studies which reported late follow-up outcomes, the overall mortality rates (Table 1) were 22.1% (205/928) in the TAVR group and 16.9% (162/958) in the SAVR group (OR 1.45, 95% CI 1.00–2.11, *I*^2^ = 50.3%) (Figure 2C).

Sensitivity analysis after the removal of studies which included patients with previous aortic valve replacement did not statistically alter short-term and mid-term outcomes (30-day mortality: OR 0.96, 95% CI 0.83–1.10; 1-year mortality: OR 1.10, 95% CI 0.77–1.56). The pooled OR for overall mortality became insignificant (OR 1.21, 95% CI 0.93–1.58). However, the trend toward increased mortality with TAVR was still observed.

### Post-procedural Complication

**Figure 3:**
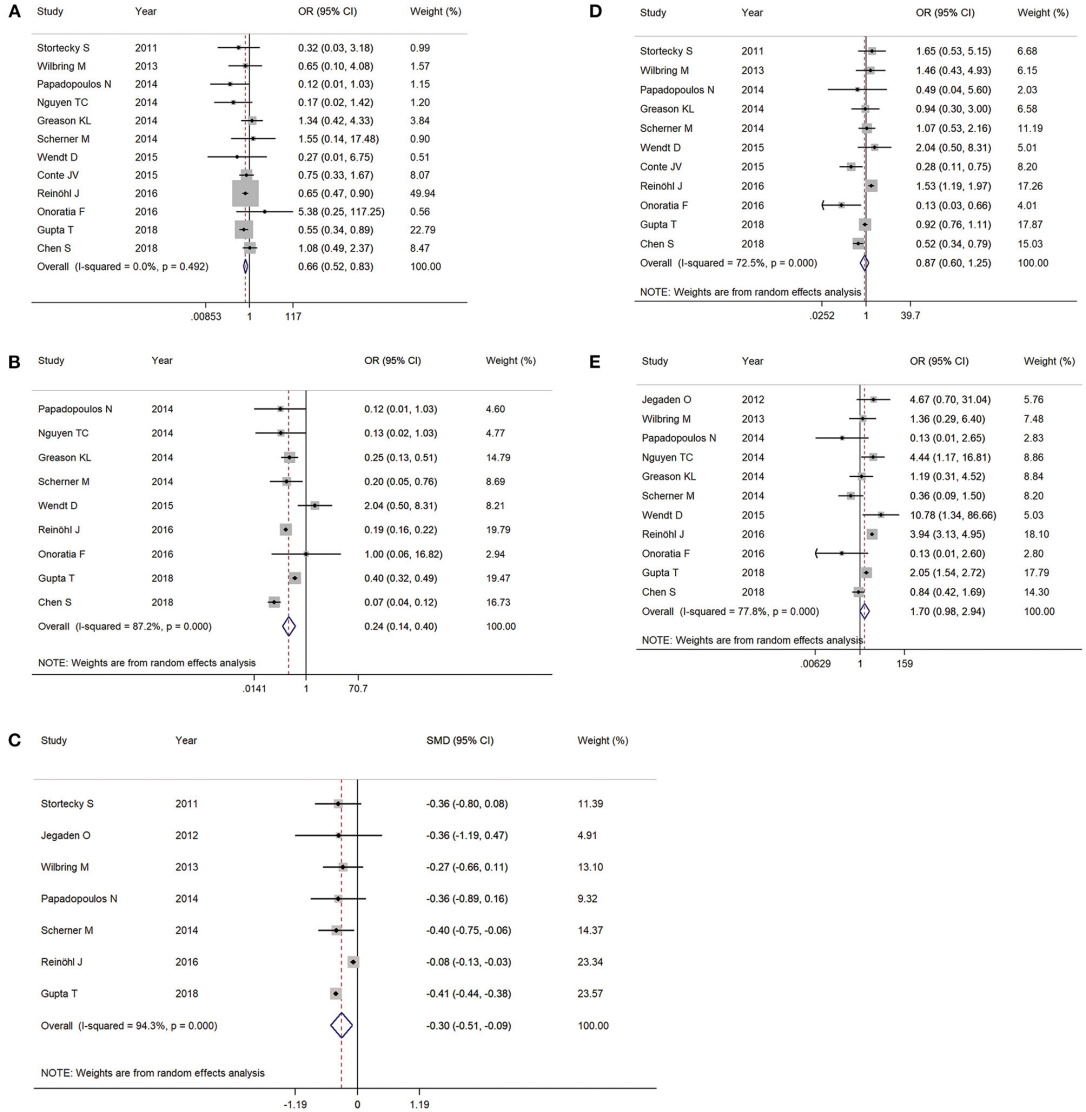
The meta-analysis for post-procedural complications as **(A)** stroke, **(B)** bleeding (major or worse), **(C)** length of stay, **(D)** acute kidney injury, and **(E)** permanent pacemaker implantation.

PCS patients undergoing TAVR was associated with a lower rate of stroke (OR 0.66, 95% CI 0.52–0.83, *I*^2^ = 0%) (Figure 3A), bleeding (major and life-threatening bleeding) (OR 0.24, 95% CI 0.14–0.40, *I*^2^ = 87.2%) (Figure 3B), and shorter length of hospital stay (standardized mean difference −0.30, 95% CI −0.51–0.09, *I*^2^ = 94.3%) (Figure 3C). However, there was no statistical significance with acute kidney injury (OR 0.87, 95% CI 0.60–1.25, *I*^2^ = 72.5%) (Figure 3D) or PPMI (OR 1.70, 95% CI 0.98–2.94, *I*^2^ = 77.8%) (Figure 3E) between the two groups.

### Subgroup Analysis by Surgical Risk Classification

**Figure 4:**
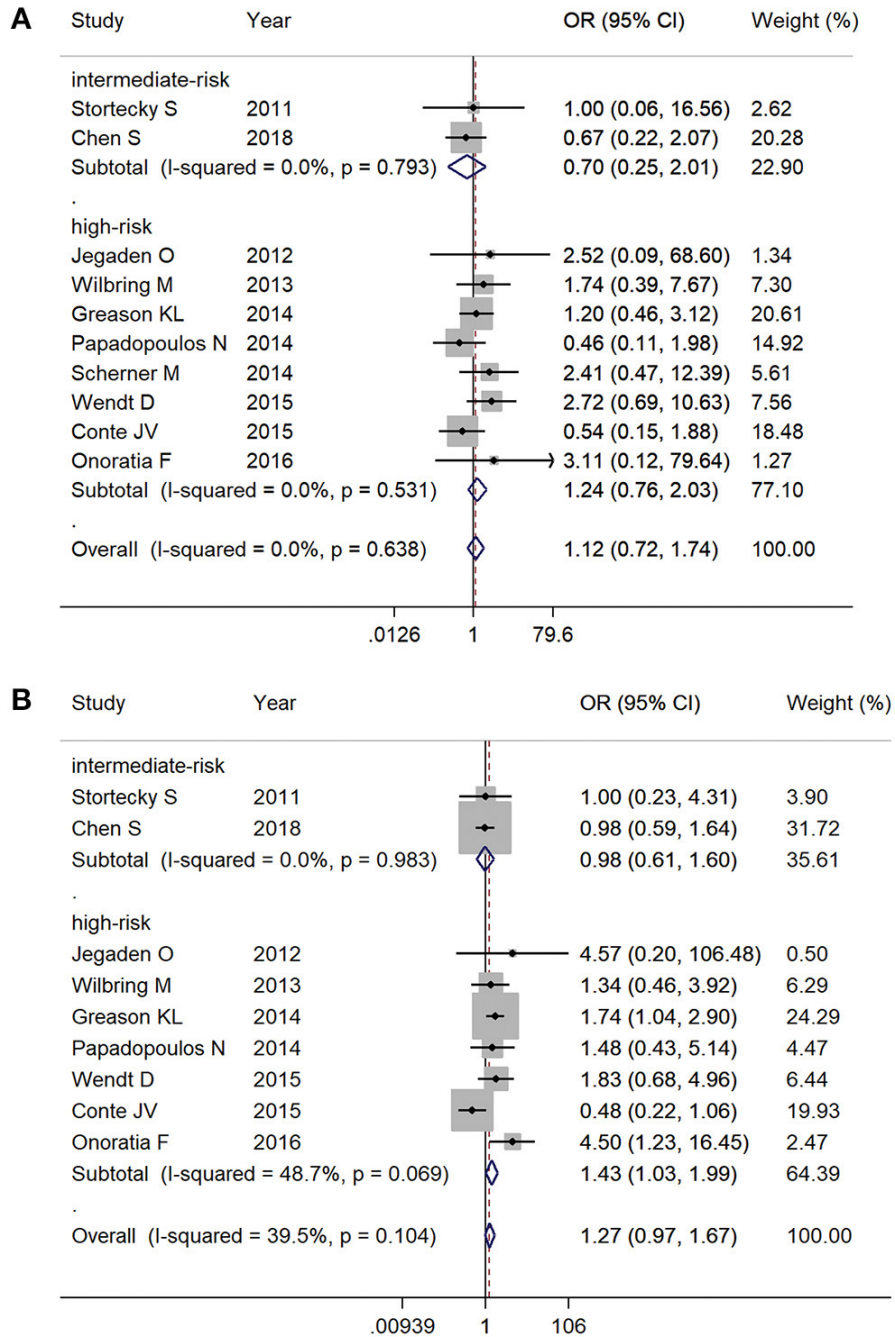
Subgroup analysis by surgical risk classification: **(A)** 30-day mortality and **(B)** overall follow-up mortality.

At 30-day follow-up, there was no significant mortality difference between TAVR and SAVR groups in both intermediate-risk (2.1% [6/285] vs. 2.9% [9/304], respectively; OR 0.70, 95% CI 0.25–2.01, *I*^2^ = 0%) and high-risk subgroups (7.3% [39/536] vs. 5.9% [29/492], respectively; OR 1.24, 95% CI 0.76–2.03, *I*^2^ = 0%) (Figure 4A). Overall follow-up mortality for both TAVR and SAVR was comparable among those with intermediate risk (12.6% [36/285] vs. 12.8% [39/304], respectively; OR 0.98, 95% CI 0.61–1.60, *I*^2^ = 0%). For high-risk patients, however, TAVR was found to be associated with higher mortality rate compared with SAVR (23.5% [108/459] vs. 17.8% [57/433], respectively; OR 1.43, 95% CI 1.03–1.99, *I*^2^ = 48.7%) (Figure 4B).

### Subgroup Analysis by Access

**Figure 5:**
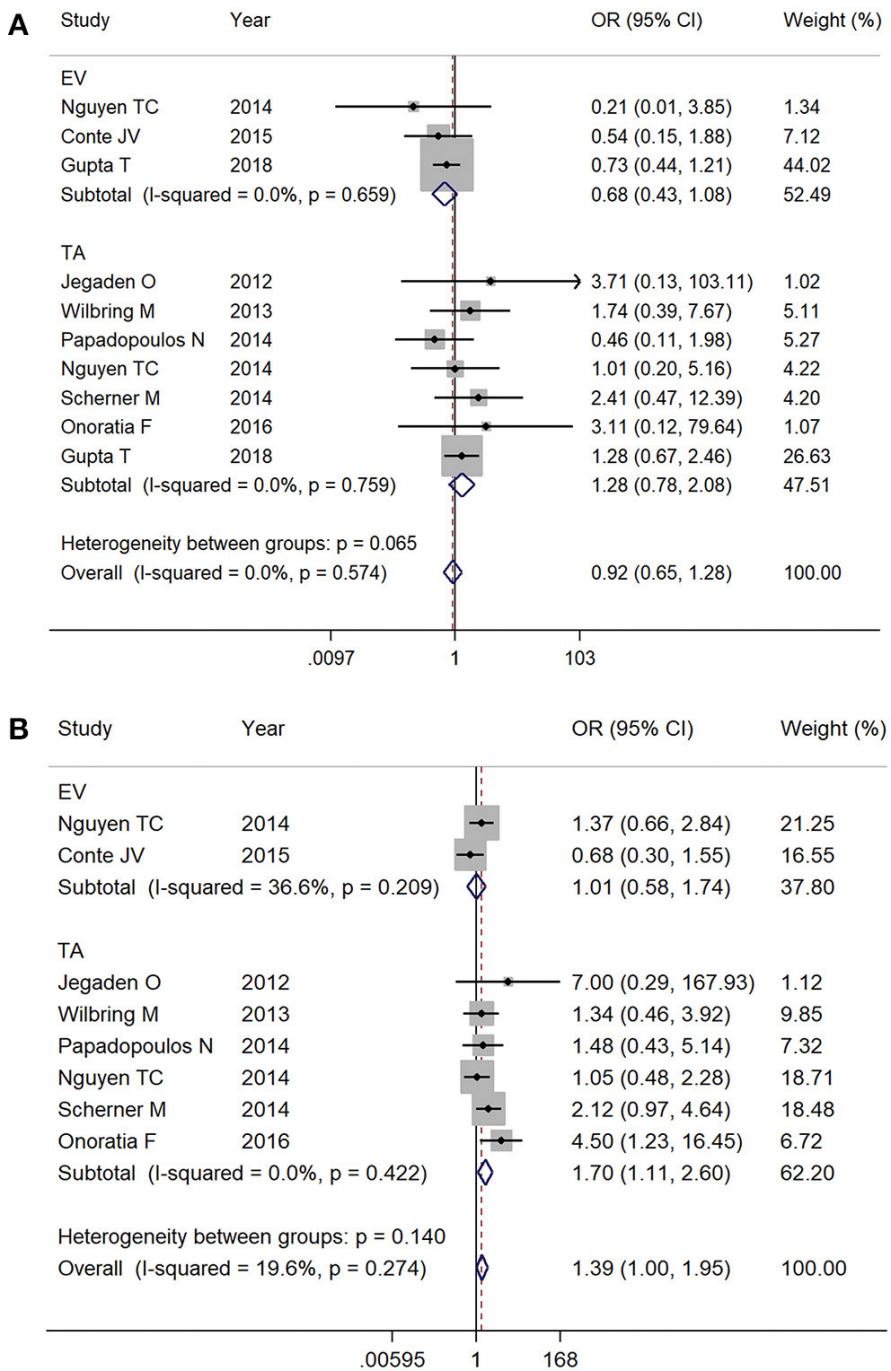
Subgroup analysis by access: **(A)** 30-day mortality and **(B)** overall follow up mortality. EV, endovascular; TA, transapical.

Of the included studies, there are 380 cases of TF TAVR and 2,211 cases of TA TAVR, with the rest of the cases being EV TAVR but without specifications of the access artery or TAVR without specifications of access route. We delineated mortality by EV TAVR and TA TAVR separately from available data. The EV (1.9% [137/7,171] vs. 2.6% [167/6,429]; OR 0.68, 95% CI 0.43–1.08, *I*^2^ = 0%) and TA (3.7% [78/2,136] vs. 2.6% [171/6,508]; OR 1.28, 95% CI 0.78–2.08, *I*^2^ = 0%) (Figure 5A) subgroups both showed comparable mortality outcomes with SAVR at 30-day follow-up. During the entire follow-up period, there was no significant mortality difference between EV TAVR and SAVR group (15.1% [25/166] vs. 18.5% [47/259], respectively; OR 0.68, 95% CI 0.43–1.08, *I*^2^ = 0%). However, patients who underwent TAVR with the TA approach were associated with higher mortality rate compared with SAVR (22.9% [41/179] vs. 17.2% [48/279], respectively; OR 1.70, 95% CI 1.11–2.60, *I*^2^ = 0%) (Figure 5B).

### Analysis by Prostheses Type

Three studies reported the prostheses type, with the PARTNER trials using balloon-expandable valve and CoreValve High Risk (CHR) study using self-expandable valve. Pooled subgroup analysis could not be performed due to the limited number of patients, but we compared the study endpoints among TAVR procedures with different prosthesis types. According to the PARTNER 1A and 2A trial, the 1-year all-cause mortality in TAVR patients with PCS was 25.0% (6) and 18.6% (7), respectively. In CHR study, 1-year all-cause mortality in TAVR patients with PCS was 8.8% (8).

### Multivariate Analysis of Outcomes

There was no significant difference between TAVR and SAVR groups in both 30-day and overall mortality by multivariate analysis. However, the risk of TAVR group showed an increased trend over time during follow-up (adjusted OR 30 days: 0.62, 95% CI 0.36–1.06, *I*^2^ = 38.5%; adjusted HR follow-up results: 1.20, 95% CI 0.93–1.56, *I*^2^ = 28.4%) (Supplementary Figures 1A,B).

## Discussion

The principal findings of the current meta-analysis consist of the following: (1) both TAVR and SAVR demonstrated similar short- and mid-term mortality outcomes in patients with PCS; (2) TAVR was found to be associated with reduced risk of post-procedural complications including stroke, bleeding, and decreased hospital-stay duration; (3) TA TAVR patients who were at high-risk had higher late follow-up mortality rate compared with SAVR; and (4) EV TAVR patients had comparable mortality outcome with SAVR.

Many patients who have had TAVR or SAVR have had PCS. In the SURTAVR (19) or PARTNER IIA (3) trials, the rate of PCS patients was ~25%. Before TAVR was commercially available, re-operative SAVR had been the primary treatment option. The reported peri-operative mortality for re-do SAVR ranged from 4 to 7% in individual studies (20–22), and long-term prognosis was generally good with a survival rate of 80–90% at 3-year follow-up (22, 23). The safety and feasibility of SAVR was confirmed in previous publications. However, the technical challenges of re-operative SAVR are well-known, which includes repeat sternotomy, risk of scarring of the pleura, and damage to the bypass arteries (24). Recent published studies from the US National Inpatient Sample (18, 25) and a large German registry (16) have demonstrated that the rate of TAVR performed in PCS patients has increased dramatically in recent years (in both studies, *P*_*trend*_ < 0.001). Therefore, understanding and predicting which patients are more suitable for either TAVR or SAVR in these cases is clinically prominent.

Two previous meta-analysis demonstrated that patients with prior coronary artery bypass graft (CABG) undergoing TAVR had a similar risk of stroke and 1-year mortality compared with SAVR (26, 27). However, these studies only included five (*n* = 872) and seven studies (*n* = 1,121), respectively. In addition, subgroup analysis with intermediate risk cohort or specific access route was omitted. Furthermore, due to the inherited study limitation at the time, the author did not perform outcome multivariate analysis. In the current study, TAVR had comparable outcomes to SAVR. This result is similar to several previous published studies (6, 7, 9, 11, 16, 18), but the reported rate of stroke and bleeding (major and worse) were observed to be significantly lower in the TAVR group compared with SAVR. In those who require re-do surgery, scarring, adhesion, calcification, and fibrosis are often present, and may increase the risk of embolization. From the STS database, the overall incidence of stroke after isolated SAVR was ~1.5% (28). In the current study, however, this rate was much higher in patients with PCS (3.8% in current study, while 2.4% in TAVR group). Nevertheless, it was reported that this factor did not influence short-term mortality (29). As expected, TAVR was found to be associated with a reduction in bleeding complications compared with SAVR, and PCS did not seem to increase these risks. Reinöhl et al. (16) also demonstrated that bleeding events in (TAVR vs. SAVR) have declined over the years, which is likely due to improvement of operator experience and valve system technologies over the years, thus, for the resulting trend. Other benefits of TAVR included shortened post-procedural hospitalization and lower overall medical cost (30). Another trend we observed in the (TAVR group vs. SAVR) was the higher rate of PPMI, which is in line with several previously published literatures (19, 31). It is worthy to note that the rate of PPMI in TAVR patients has decreased significantly in recently published studies and is likely due to the release of new-generation valves in recent years. The rate of PPMI in the new generation device was reported to be decreased by up to 7–13% vs. the older generation valves (32, 33).

During subgroup analysis, higher baseline operative risk score and TA access was found to be significantly associated with increased risk of overall follow-up mortality. This discrepancy between high-risk and moderate-risk patients was also reported in the famous PARTNER studies (1, 3). An important factor for this trend might be due to the fact that higher-risk patients are often associated with multiple baseline comorbidities. As well as that, the results of the other non-matched analysis studies included shows that the mean age and the predicted risk of mortality were much higher in TAVR group (15, 16). Therefore, TAVR patients in these studies were more likely to be exposed to baseline risk factors, thus, resulting in potential baseline confounding. Nevertheless, the risk of bias assessment in the current study indicates there was no bias in the primary overall survival assessment. Moreover, results from previous studies have shown that patients receiving transapical access have a higher rate of peripheral artery disease, which hinders the ability to deploy transfemoral access (2). Additional risk factors such as diabetes mellitus, hypertension, and previous history of stroke/transient ischemic attack may have also contributed to the higher mortality in TA TAVR (2, 34). Meanwhile, TF TAVR is favored in elderly patients who are at increased risk for surgery and have proven to be non-inferior, even superior to SAVR (1–3, 35). However, there were no significant differences in Log EuroSCORE and STS score between EV and TA TAVR in the included studies. Lastly, according to the studies based on PARTNER and CHR trials (6–8), self-expandable valves may be associated with lower mortality and post-operative complications than balloon-expandable valves in patients with PCS. However, due to the limited number of patients in these studies, further investigation is required to determine the efficacy of self- vs. balloon-expandable valves in patients with PCS.

Another important finding of our study was that in patients with PCS, the all-cause mortality was lower in EV TAVR than SAVR and TA TAVR, which was in agreement with the results of previous randomized controlled trials (3, 36). Similar conclusions were also drawn by other observational studies and meta-analyses (37, 38). Compared with SAVR or TA TAVR, TF TAVR is able to avoid the scar tissue and adhesions in the thoracic cavity caused by previous surgical interventions, thus improving its safety and efficacy in patients with PCS. Recently published guidelines on valvular heart disease indicated that TAVR with the TA approach was an alternative choice to TF TAVR when the anatomy of the individual femoral arteries were deemed inaccessible (2). This possible inferiority outcome results with TA TAVR over SAVR and TF TAVR in PCS patients in the current study are in line with prior published literature. With the expansion of the clinical indications of TAVR procedures into low-risk populations, endovascular (especially transfemoral) access of TAVR has become the treatment of choice in a widening series of clinical scenarios due to its minimally invasive incisions compared with traditional thoracotomy (36). In addition, with the continuous optimization of TAVR techniques, smaller sheath size as well as more flexible deployment device has enabled the establishment of access route in patients with complex cardiovascular anomalies such as atherosclerosis and anatomical narrowing or tortuosity of the major arteries. As the application of TF TAVR continues to expand in patients of various risk scores and comorbidities, the safety of TF TAVR in patients with PCS requires a more carefully designed larger study with a longer follow-up duration.

### Study Limitations

The main limitation of this study is that these results should be interpreted with caution as meta-analyses are not designed to give definitive answers or address issues at patient baseline level. Second, the number of EV TAVR was much smaller compared with TA TAVR, especially the TF access. Third, although we performed multivariate analysis on follow-up mortality, the various adjusted models conducted may have resulted in confounding. Fourth, due to the limitation of the number of studies enrolled, and none of the studies were randomized control trials, we could not perform a network meta-analysis to make indirect comparisons. Fifth, the included studies are real-world cohort studies, and apart from the PARTNER and CoreValve studies, most of them did not specify the types and sizes of the prostheses used. Thus, we could not perform a subgroup analysis regarding various prostheses. Lastly, due to the lack of studies which reported the type of PCS surgeries the patients has had, subgroup analysis with specific type of PCS was omitted.

## Conclusion

Patients with PCS undergoing TAVR have similar short-term mortality compared with SAVR. However, the incidence of stroke and bleeding complications were observed to be lower in the TAVR group. Those with high-risk or undergoing TAVR *via* TA access were associated with higher mortality compared with SAVR. Surgical risk assessment and access route selection in patients with PCS require careful consideration.

## Data Availability Statement

The original contributions generated for the study are included in the article/Supplementary Material, further inquiries can be directed to the corresponding author/s.

## Author Contributions

All authors listed have made a substantial, direct and intellectual contribution to the work, and approved it for publication.

## Conflict of Interest

The authors declare that the research was conducted in the absence of any commercial or financial relationships that could be construed as a potential conflict of interest.

## Abbreviations

PCS: prior cardiac surgery
TAVR: transcatheter aortic valve replacement
SAVR: surgical aortic valve replacement
SMD: standardized mean difference
CHR: CoreValve High Risk
AS: aortic stenosis
TA: transapical
STS: Society of Thoracic Surgeons
PPMI: permanent pacemaker implantation
EV: endovascular
CABG: coronary artery bypass graft.
