# Supplementary material for: Transcatheter and Surgical Aortic Valve Replacement in Patients With Previous Cardiac Surgery: A Meta-Analysis
Source: Front Cardiovasc Med. 2021 Feb 10;7:612155. doi: 10.3389/fcvm.2020.612155 (PMC7902485; doi:10.3389/fcvm.2020.612155)
Supplement: Supplementary file 1 [file Data_Sheet_1.docx]

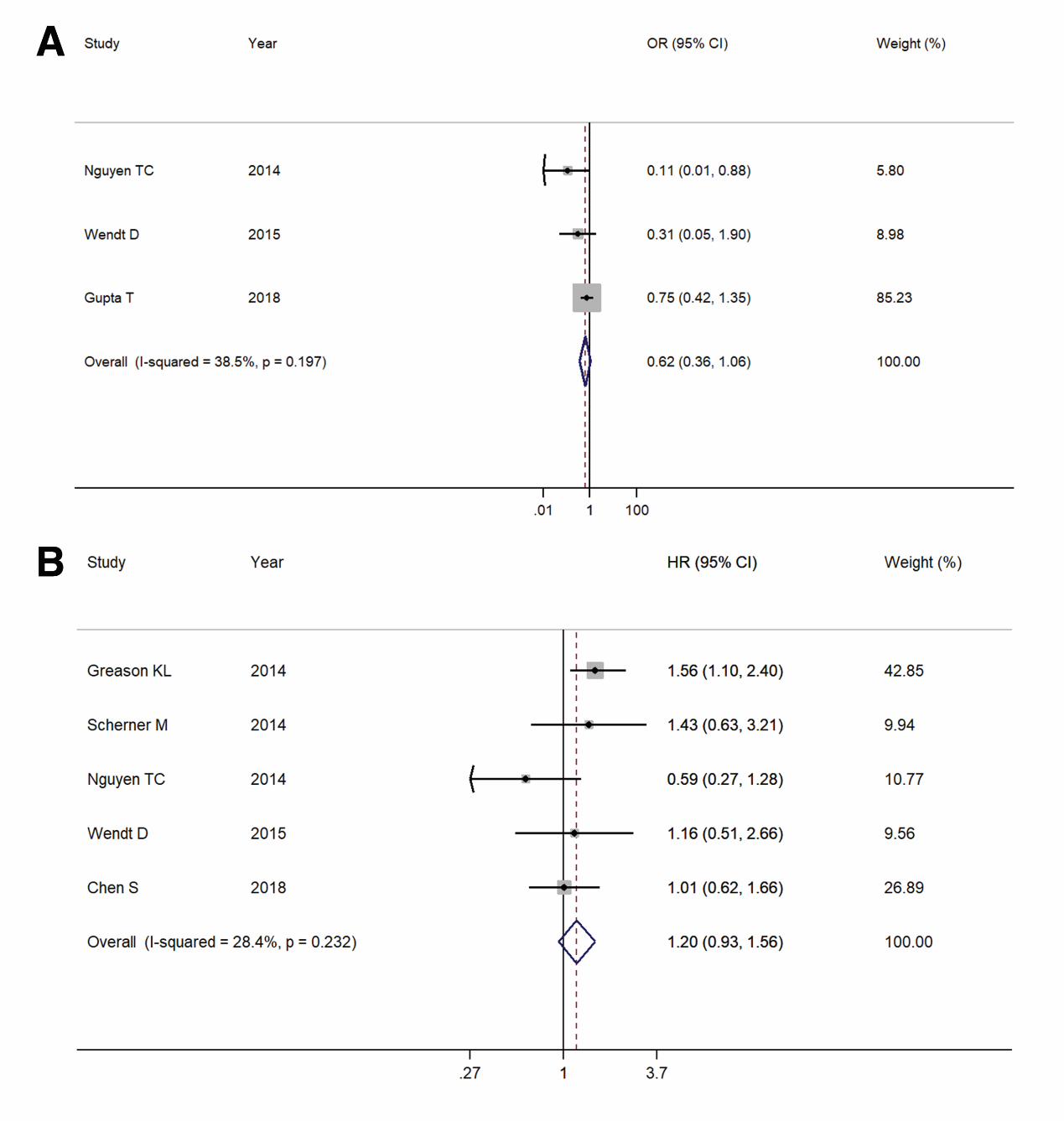


Supplementary Figures 1A,B: Multivariate analysis of TAVI treatment on all-cause mortality: A)30 days and B) follow up period. TAVR: transcatheter aortic valve replacement
